# Seasonal differences in breastfeeding in the United States: a secondary analysis of longitudinal survey data

**DOI:** 10.1186/s13006-022-00479-4

**Published:** 2022-07-07

**Authors:** Claudia W. Strow, Brian K. Strow

**Affiliations:** Rinker School of Business, Palm Beach Atlantic University, Florida, USA

## Abstract

**Background:**

Both the consumption of breastmilk in infancy and a person’s season of birth influences his or her health, educational, professional, and behavioral outcomes. Further, season of birth effects differ by sex. However, current research, for the most part, neglects to examine if season of birth and breastfeeding are related. This paper examines the impact of sex-based variations in season of birth on breastfeeding likelihood and duration in the U.S.

**Methods:**

Using data from children born to female respondents of the National Longitudinal Survey of Youth 1979 (born between 1970 and 2012), this study examines with Probit, Negative Binomial, and Ordinary Least Squares (OLS) regressions if a child’s season of birth and sex are correlated with breastfeeding incidence and duration. The breastfeeding incidence and duration data are self-reported by the mother.

**Results:**

Season of birth has a small but statistically significant impact on the incidence and duration of breastfeeding, which varies depending on the sex of the infant. Mothers giving birth to sons in the spring are 13.5% less likely to breastfeed than those giving birth to sons in the winter (with a *p* - value of 0.0269). Mothers with daughters born in the summer or fall (autumn) breastfeed slightly longer than mothers with daughters born in the spring. On average, mothers of summer-born daughters breastfeed 4.1% longer (with a 95% confidence interval of 0.3 - 7.8) and those with fall-born daughters 3.8% longer (with a 95% confidence interval of 0 - 7.5). Mothers giving birth to daughters in the spring are also significantly less likely to reach the breastfeeding six-week duration target (compared to fall and winter births) and the one-year duration target (compared to fall births).

**Conclusions:**

These findings suggest that the costs and benefits of breastfeeding an infant vary with the season of birth and the sex of the child. This finding could explain some of the season of birth effects previously identified in the literature. Further, policymakers seeking to increase breastfeeding rates should consider the reduced breastfeeding rates and durations for children born in the spring.

## Background

There is strong evidence regarding the existence of season of birth variations in health, personality, and even IQ [1–10]. Similar variations in health, personality, and IQ exist based on an infant’s feeding method [11–13]. This paper uses data from the United States to examine if an infant’s season of birth affects his or her feeding method. While existing research notes the presence of seasonal trends in breastfeeding within developing nations, researchers have yet to fully investigate the possibility of seasonal differences in breastfeeding within developed nations. If seasonal differences in breastfeeding exist, then strategies and policies which influence breastfeeding incidence and duration could be structured to target the most at-risk birth seasons and could potentially help counteract some of the unfavorable effects of being born in a particular season.

## Existing research

As with any decision, a mother chooses to initiate breastfeeding (or continue breastfeeding) if the benefits outweigh the costs. A number of correlates to breastfeeding exist in the literature, and these correlates change the cost and benefit analysis in ways that influence breastfeeding decisions. For example, in the United States, more educated women likely have greater knowledge of the benefits of breastfeeding, greater access to financial resources or paid leave to support the financial costs of breastfeeding, are less likely to be recipients of the Special Supplemental Nutrition Program for Women Infant and Children (WIC) receiving discounted or free infant formula, and are more likely to have peers that breastfeed. Similar relationships exist based on a woman’s marital status, income, labor force participation, age, family size, generation, and potentially race [14–18].

We suspect that a child’s season of birth and sex are also correlated with the costs and benefits of breastfeeding. For instance, mothers wishing to improve health outcomes should be more likely to breastfeed in colder seasons when infections are more common, and the benefit of increased immunity is more important. The costs and benefits of continuing to breastfeed may also differ by season or sex due to fertility concerns. Breastfeeding durations may be shorter for daughters if there is a preference for sons, due to breastfeeding’s contraceptive effect [19]. For some ethnicities, female infants have a greater likelihood of being breastfed and are breastfed for longer durations than male infants [20]. In a similar vein, breastfeeding patterns could vary by season if there is a preferred time of the year to become pregnant or give birth [21].

While several existing studies examine seasonal and sex-based differences in breastfeeding incidence, little attention is given to the two combined. Furthermore, the vast majority of existing seasonal studies consider nations such as Tanzania, Egypt, India, Brazil, Kenya, and Senegal that are less developed or have climates with only two seasons: wet and dry [22]. These studies document numerous seasonal effects on breastfeeding including: increased weaning in warmer months or following a rainy season, decreased breastfeeding rates during hot weather (as pregnancy rates climb), decreased duration in the months leading up to winter, and longer breastfeeding durations of malnourished children [23–26].

Samuelsson and Ludvigsson present one of the only seasonal breastfeeding studies of a developed nation [27]. They investigate if birth season and breastfeeding differences in Sweden could explain the elevated risk of summer-born children developing diabetes mellitus. They find that summer-born babies are exclusively breastfed for a shorter duration; however, they find no significant difference in breastfeeding duration between those developing diabetes and the control group. Yet, children born in the summer in their study are both more likely to develop diabetes and are breastfed for shorter durations. The only other examination of seasonal breastfeeding in a developed nation is a letter to the editor by the present authors, where we summarized some preliminary findings of our research [28].

Not only might feeding patterns differ by season, evidence suggests that breast milk quality (including complex nutrients for gut and brain health and disease prevention) changes in regard to both the season and the child’s gender [29, 30]. In fact, Kanazawa and Segal explore health outcomes of same sex versus opposite-sex twins from the U.S. and find that opposite-sex twins appear to be disadvantaged in growth, presumably from the lack of sex specificity in their breast milk [31].

Given that consuming breastmilk in infancy has been linked to health and educational benefits, and that breast milk content differs by sex and potentially season, it is surprising that few researchers explore the implications of seasonal variation in breastfeeding in developed nations. Furthermore, given individuals’ different lifestyles in cold versus warm months and differences in virus exposure, as well as potential preferences for a subsequent son or a daughter, it is reasonable to expect that women may choose to breastfeed at a different rate or for a different duration based on the season in which they are giving birth and the sex of the child. This paper fills the void in the current literature by exploring whether one’s season of birth affects his or her likelihood of being breastfed and the duration of said breastfeeding in the United States.

## Methods

This study uses data for children born to female respondents in the National Longitudinal Survey of Youth from 1970 to 2012 [1, 32]. Conducted by the Bureau of Labor Statistics since 1979, the National Longitudinal Survey of Youth annually or biannually interviews 12,686 individuals from the United States who were 14 to 21 years of age as of December 31, 1978 [1]. For female respondents with children, the survey collects information from mothers about the children’s infant-feeding methods and durations, behaviors, outcomes, and socioeconomic backgrounds [33]. Our study sample uses the merged mother-child survey and includes 6877 children born to women from the original sample as of 2012. By 2012, most of the women in the study had likely completed childbearing, as the women were 47-54 years of age.

This survey allows us to observe seasonal variations in breastfeeding duration and incidence. Furthermore, it allows us to control for factors likely correlated to both season of conception and breastfeeding decisions, such as race, education, labor force participation, income, marital status, maternal age, family size, and child birth order. The breastfeeding data in the survey are self-reported information from the mother at the first survey following the birth of the child (with the question initially being asked in the 1983 survey). The question is asked of the mother “When (child) was an infant, did you breastfeed (him/her) at all?” The follow-up question for those responding “yes” is “How many weeks/months old was (he/she) when you quit breastfeeding (him/her) altogether?” [33]. All answers to this question are converted to weeks.

For our examination, we define winter births as those occurring in December, January, or February. Spring births occur in March, April, or May, while summer births occur in June, July, or August. September, October, or November births are classified as fall births. The socioeconomic controls include race, mother’s highest degree ever reported in the survey, and ASVAB (Armed Services Vocational Aptitude Battery) scores in math. ASVAB scores are used as a proxy for IQ [34]. We also control for the mother’s marital status at the time of the baby’s birth, birth order, the number of kids the mother listed in 1979 as ideal for a family size, the total number of children ever born to the mother, and the age of the mother at her first birth.

We also control for the age group (10-19, 20-29, or 30 +) of the mother at this particular child’s birth. We control to the best of our ability for the number of hours worked by the mom in the year (or year prior) to the child’s birth and the total net family income from 1 to 2 years prior to the child’s birth. Lastly, we use dummy variables for the decade of birth in order to control for cultural changes in breastfeeding that transpired from the 1970’s to today.

To examine the impact of season of birth on breastfeeding incidence and duration, we run estimations for the sample as a whole and for the male and female samples separately. We use the SAS programming language to estimate incidence using ordinary least squares (OLS) and estimate duration using a negative binomial model. We test the significance of the OLS findings by also running probit models. The probit models yield the same findings for significance as the OLS models, thus for ease of interpretation, only the OLS results are presented here. Lastly, we test the impact of season of birth and sex on one’s likelihood of reaching breastfeeding duration targets of 6 weeks (often the initial goal set by mothers), and the targets of 6 months, and 1 year commonly identified by the American Academy of Pediatrics [35, 36].

## Results

Table 1 lists sample means. In our sample, 44.6% of children breastfeed, while the average duration of breastfeeding is 21.4 weeks. The birth seasons in our sample are somewhat evenly distributed, with the largest percentage of babies being born in the summer (26.6%) and the fewest being born in the spring (23.9%).

**Table 1:** Descriptive statistics

	Total N	All Means	Male N	Male Means	Female N	Female Means
**General breastfeeding measures**						
Breastfed (y/n)	6600	0.445909	3388	0.436836	3212	0.45548
Total duration (In weeks)	2784	21.39332	1408	21.6108	1376	21.17078
**Duration targets (y/n)**						
6 weeks	2784	0.700072	1408	0.697443	1376	0.702762
6 months	2784	0.282687	1408	0.291193	1376	0.273983
1 year	2784	0.077227	1408	0.081676	1376	0.072674
**Season of birth**						
Winter	6877	0.24342	3522	0.249574	3355	0.23696
Spring	6877	0.239494	3522	0.229983	3355	0.249478
Summer	6877	0.265668	3522	0.266326	3355	0.264978
Fall	6877	0.251418	3522	0.254117	3355	0.248584
**Race/ethnicity**						
Non-black/non-Hispanic	6877	.452232	3522	0.454571	3355	0.449776
Black	6877	0.332122	3522	0.327371	3355	0.337109
Hispanic	6877	0.215646	3522	0.218058	3355	0.213115
**Education level**						
High school dropout	6876	0.128126	3521	0.131213	3355	0.124887
High school	6876	0.502909	3521	0.499006	3355	0.507005
Associates	6876	0.118819	3521	0.118432	3355	0.119225
Bachelors	6876	0.123473	3521	0.123544	3355	0.123398
Graduate	6876	0.055701	3521	0.055098	3355	0.056334
Other degree	6876	0.070972	3521	0.072707	3355	0.069151
**Test score**						
ASVAB math score	6490	3562.08	3329	3621.49	3161	3499.51
**Age of mother at birth**						
10–19	6877	0.184964	3522	0.186542	3355	0.183309
20–29	6877	0.577287	3522	0.580068	3355	0.574367
30+	6877	0.23775	3522	0.23339	3355	0.24232
**Mother’s lifestyle factors at the birth of the child**						
Hours worked for pay	5930	1066.27	3025	1051.45	2905	1081.69
Married (y/n)	6877	0.697688	3522	0.698183	3355	0.697168
**Birth order of the child**						
Firstborn	6877	0.431002	3522	0.429869	3355	0.432191
Secondborn	6877	0.320634	3522	0.327371	3355	0.313562
Other	6877	0.248364	3522	0.24276	3355	0.254247
**Projected number of children**						
Ideal family size desired by the mother as a teen	6848	2.591268	3510	2.583191	3338	2.59976
**Number of kids ever born**						
Two children	6877	0.332994	3522	0.337876	3355	0.327869
Three children	6877	0.300858	3522	0.30494	3355	0.296572
Four children	6877	0.160535	3522	0.160136	3355	0.160954
5+ children	6877	0.124618	3522	0.116695	3355	0.132936

The covariates of breastfeeding exhibit the impacts predicted by prior studies. For instance, breastfeeding incidence is lower for black infants and Hispanic female infants, is lower for those whose mothers had less than a college education, and is lower for those whose mothers scored lower on the ASFAB and for those whose mothers worked more hours. Likewise, breastfeeding incidence is lower for infants born into lower income classes, those with a younger mother, and for infants whose parents are not married at birth. Women with a smaller desired family size (reported when they were a teenager) were less likely to breastfeed.

In terms of seasonal differences, at first glance, and without any controls for socioeconomic factors, there appears to be little seasonal difference in breastfeeding incidence. However, once we include controls for socioeconomic factors, we find that season is a significant determinant of breastfeeding incidence for sons (Table 2). In particular, we find that mothers with sons born in the winter are 13.5% more likely to breastfeed than those with sons born in the spring (Table 3). The breastfeeding incidence of daughters does not appear to be significantly related to season of birth.

**Table 2:** Breastfeeding incidence

	All	Male	Female
**Intercept**	− 0.08281 (0.10016)	−0.04778 (0.13778)	− 0.17597 (0.14558)
**Sex**			
Male	−0.029919** (0.01332)		
**Season of birth**			
Summer	−0.00638 (0.01862)	0.01771 (0.02630)	−0.02615 (0.02648)
Fall	0.01486 (0.01911)	0.04673 (0.02697)	−0.01134 (0.02722)
Winter	0.02413 (0.01904)	0.05898** (0.02663)	−0.00299 (0.02742)
**Race/ethnicity**			
Black	−0.15778*** (0.01900)	−0.15603*** (0.02694)	− 0.15872*** (0.02693)
Hispanic	0.02463 (0.01911)	−0.01687 (0.02655)	0.07047*** (0.02766)
**Education level**			
High school	0.06315*** (0.02426)	0.07253** (0.03362)	0.06075 (0.03516)
Associate	0.08972*** (0.03024)	0.09373** (0.04192)	0.09388** (0.04389)
College	0.17706*** (0.03251)	0.18069*** (0.04528)	0.17989*** (0.04689)
Graduate school	0.12838*** (0.03814)	0.15141*** (0.05341)	0.12364** (0.05490)
Other degree	0.09544*** (0.03423)	0.12936*** (0.04638)	0.06717 (0.05106)
**Test score**			
ASVAB math	0.00002909*** (0.00000329)	0.00003529*** (0.00000459)	0.00002312*** (0.00000473)
**Mother’s lifestyle factors at the birth of the child**			
Hours worked for pay	−0.00001909** (0.00000826)	− 0.00002505** (0.00001141)	− 0.00001659 (0.00001203)
Income	−2.07899E−9 (8.716857E-8)	6.977359E-8 (1.135965E-7)	-9.3693E-8 (1.36012E-7)
Married at birth	0.12916*** (0.01767)	0.11193*** (0.02487)	0.14640*** (0.02525)
Age of Mother at first birth at first birth	0.01277*** (0.00228)	0.00834*** (0.00317)	0.01748*** (0.00328)
**Age of mother at birth**			
10–19	−0.14574*** (0.03765)	−0.16846*** (0.05308)	− 0.11983** (0.05354)
20–29	−0.06302*** (0.02413)	− 0.07647** (0.03413)	−0.04370 (0.03420)
**Birth order of the child**			
Firstborn	0.06680*** (0.02162)	0.11528*** (0.03044)	0.01390 (0.03081)
Youngest	−0.01997 (0.02065)	− 0.02675 (0.02883)	− 0.01227 (0.02971)
**Projected number of children**			
Ideal family size desired as a teen	0.01716*** (0.00455)	0.02229*** (0.00642)	0.01155 (0.00648)
**Number of kids ever born**			
Two children	0.04167** (0.03234)	0.08329 (0.04556)	0.00623 (0.04615)
Three children	0.08172*** (0.03743)	0.11874** (0.05245)	0.05382 (0.05367)
Four children	0.12557*** (0.04236)	0.16888*** (0.05927)	0.08925 (0.06083)
5+ children	0.12147*** (0.04670)	0.16233*** (0.06572)	0.08668 (0.06662)
**Decade of child’s birth**			
1970’s	−0.01661 (0.06130)	−0.11029 (0.08628)	0.07577 (0.08751)
1980’s	−0.01837 (0.05019)	−0.07025 (0.07039)	0.03615 (0.07188)
1990’s	−0.00959 (0.04550)	−0.06460 (0.06390)	0.04888 (0.06502)
**Regression statistics**			
Adjusted *R*^2^	0.2240	0.2330	0.2174
n	4400	2251	2149

Standard errors from ordinary least squares estimations are in parentheses

***Significantly different from 0 at the 99% level, and ** at the 95% level

**Table 3:** Percentage change in season of likelihood compared to spring births

	***All***	***Male***	***Female***
Summer	−1.43%	4.05%	−5.74%
Fall	3.30%	10.70%	2.49%
Winter	5.41%	13.5%**	−0.66%

**Denotes significantly different from 0 at the 95% level

In contrast, season of birth appears to have a small but significant impact on the breastfeeding duration of daughters, but has no significant effect on the sons’ durations (Table 4). Mothers with daughters born in the summer or fall breastfeed longer than mothers with daughters born in the spring (on average 4.1% longer for summer-born daughters and 3.8% longer for fall-born daughters). Thus, spring-born males are less likely to breastfeed while spring-born daughters breastfeed for a slightly shorter duration than their winter and fall-born counterparts. Decade of birth has a major impact on breastfeeding duration, but appears to have no statistically significant impact on incidence. For males, the largest factor driving breastfeeding duration appears to be the number of children in the family. Male children are breastfed approximately 8-18% longer if they are in household with more than one child.

**Table 4:** Breastfeeding duration estimated using a negative binomial model

	All	Male	Female
**Intercept**	4.9966 (7.3766)	−0.3997 (10.2272)	10.5373 (10.6594)
**Sex**			
Male	−0.1215 (0.9719)		
**Season of birth**			
Summer	1.5145 (1.3689)	−1.5044 (1.9895)	4.0767** (1.8889)
Fall	1.9723 (1.3865)	0.1215 (2.0043)	3.7705** (1.9278)
Winter	1.9264 (1.3812)	0.4624 (1.9652)	3.1146 (1.9554)
**Race/ethnicity**			
Black	−3.1933** (1.5553)	−3.4831 (2.2455)	−2.9703 (2.1645)
Hispanic	−1.3716 (1.3344)	−0.1747 (1.9087)	−2.2999 (1.8707)
**Education level**			
High school	1.5619 (2.3159)	1.1650 (3.4197)	1.5911 (3.1691)
Associate	2.8234 (2.6308)	1.2178 (3.8532)	4.2818 (3.6313)
College	3.1363 (2.6817)	3.2755 (3.9200)	2.9931 (3.6988)
Graduate school	6.5491** (2.9011)	6.0407 (4.2056)	6.6130 (4.0439)
Other Degree	2.3204 (2.8535)	3.9257 (4.0243)	−0.2220 (4.1275)
**Test score**			
ASVAB math	0.0011*** (0.0002)	0.0009*** (0.0003)	0.0013*** (0.0003)
**Mother’s lifestyle factors at the birth of the child**			
Hours worked for pay	−0.0030*** (0.0006)	−0.0032*** (0.0008)	− 0.0027*** (0.0008)
Income	0.0000 (0.0000)	0.0000 (0.0000)	0.0000 (0.0000)
Married at birth	0.5815 (1.5629)	−1.2461 (2.2463)	2.5388 (2.1818)
Age of mother at first birth	0.4950*** (0.1598)	0.7094*** (0.2270)	0.2952 (0.2259)
**Age of mother at birth**			
10–19	−5.9093** (3.0554)	−5.5479 (4.4112)	−5.3483 (4.2386)
20–29	−2.6985 (1.7040)	−2.6266 (2.4916)	−2.4915 (2.3387)
**Birth order of the child**			
Firstborn	1.1044 (1.5698)	2.7261 (2.2892)	−0.7012 (2.1731)
Youngest	3.7197** (1.5473)	4.0268 (2.2004)	3.1348 (2.1969)
**Projected number of children**			
Ideal family size desired as a teen	−0.0591 (0.3337)	−0.1663 (0.4669)	0.1015 (0.4774)
**Number of kids ever born**			
Two children	3.6467 (2.3488)	8.0871** (3.4305)	−0.7376 (3.2356)
Three children	9.3003*** (2.7184)	14.3225*** (3.9210)	4.1879 (3.8208)
Four children	9.1642*** (3.0622)	13.3359*** (4.4094)	4.7484 (4.2944)
5+ children	12.8655*** (3.4816)	18.8453*** (5.0290)	7.1256 (4.8664)
**Decade of child’s birth**			
Seventies	−3.9606 (4.4815)	−4.0233 (6.5379)	−5.0121 (6.2320)
Eighties	−7.7156** (3.2527)	−7.7721 (4.5957)	−8.3248 (4.6169)
Nineties	−6.4642** (2.8224)	−7.0396 (3.9512)	−6.2624 (4.0305)
**Scale**	21.7123 (0.3402)	21.6101 (0.4771)	21.6193 (0.4808)

***Denotes significantly different from 0 at the 99% level, and ** at the 95% level

To further examine differences in duration by season of birth, we estimate the likelihood of meeting three durational targets: six-week, six-month, and one-year, conditional on breastfeeding at least 1 week (for the six-week target) and conditional on meeting the earlier duration target (for six-month and one-year targets). Table 5 lists these results.

**Table 5:** Season of birth and conditional likelihood of breastfeeding to the 6-week, 6-month, and 1-year duration targets

	All Genders	Male	Female
***Conditional Six-week Duration***			
***Summer***	0.00931 (0.02770)	−0.01209 (0.04041)	0.02784 (0.03819)
***Fall***	0.03840 (0.02805)	−0.01812 (0.04071)	0.10309*** (0.03897)
***Winter***	0.02859 (0.02794)	−0.02171 (0.03992)	0.07894** (0.03953)
***Conditional Six-month Duration***			
***Summer***	0.02852 (0.03606)	−0.02154 (0.05300)	0.08228 (0.05004)
***Fall***	−0.01387 (0.03632)	0.01374 (0.05359)	−0.03801 (0.05013)
***Winter***	0.07307** (0.03615)	0.08023 (0.05245)	0.06525 (0.05093)
**Conditional One-Year Duration**			
***Summer***	0.07981 (0.05186)	0.04022 (0.07646)	0.11531 (0.07358)
***Fall***	0.11498** (0.05361)	0.06065 (0.07561)	0.17360** (0.07908)
***Winter***	0.02050 (0.05059)	0.00203 (0.07095)	0.05839 (0.07485)

Estimations use the full model of controls, but only seasonal results are presented. The 6-month and 1-year duration targets are estimated only for those observations that met the shorter target (6- weeks and 6-months respectively). The sample of those breastfeeding at all includes 2037 observations (1026 males and 1011 females). The sample of those breastfeeding at least 6 weeks includes 728 males and 724 females. Those breastfeeding for at least 6 months includes 315 males and 290 females

***Denotes significantly different from 0 at the 99% level, and ** at the 95% level

For mothers with sons, season of birth does not significantly relate to any of the three durational targets. However, mothers with fall and winter-born daughters are more likely than those with spring-born daughters to breastfeed for 6 weeks or more. Among those breastfeeding at least 6 weeks, season of birth does not appear to impact the likelihood of reaching the six-month marker. Finally, mothers with fall-born daughters are more likely than those with spring-born daughters to reach the one-year mark of breastfeeding.

## Discussion

Why would breastfeeding practices differ according to season and gender? One such reason is that newborn girls have lower morbidity rates and are less likely to be affected by conditions such as congenital anomalies, infectious diseases, and lower respiratory infections [37]. While male infants are more susceptible to respiratory and other infections in infancy, females are more susceptible to autoimmune conditions [38]. Thus, it is feasible that mothers of sons may see more benefit to breastfeeding them when they are born in the winter, as rates of respiratory infections rise in the U.S. Consistent with this hypothesis, male children born in the winter season in this study are significantly more likely to be breastfed.

Among mothers who breastfeed their daughters, those giving birth in the fall or summer tend to breastfeed slightly longer than those giving birth in the spring. As the mean duration of breastfeeding in our sample is 21 weeks, it may be that the higher cost of breastfeeding in the summer heat leads to reduced breastfeeding outcomes for spring-born babies.

These findings indicate the personal costs to a mother of breastfeeding while it is hot outside (and more time is spent outdoors and/or traveling) may be higher. This finding is consistent with research from India that suggests that breastfeeding incidence and duration are greater during winter months: “It was found that the children for whom ≥ 3 months of exclusive breastfeeding (EBF) period fell during the winter season had significantly higher odds of receiving exclusive breastfeeding” [26]. However, Das et al. do not differentiate by sex, and their findings do not explain why the difference in duration is only found for daughters in the present study. If indeed the difference in duration was for reasons related to summer heat or differing summer lifestyle factors cutting short spring-born daughters’ breastfeeding duration, then one would expect to find similar duration differences spring-born males. Future examinations of differences in duration by sex and season of birth should explore why similar duration differences do not exist for males.

While these findings are the first of their kind and open the door to a better understanding of factors influencing both breastfeeding and seasonal differences in health outcomes, the present study does have some important limitations. Perhaps the biggest limitation of this study is that breastfeeding trends have changed dramatically since the time the data collection process began. This study attempts to control for this change using decade of birth variables, but a future study should compare these findings with those using contemporary cross-sectional data. A second limitation of this study is that it relies on a mother’s reporting of breastfeeding incidence and duration (sometimes years after the birth of the child). It also fails to quantify whether the breastfeeding is exclusive. Further, this study does not examine the health outcomes as they relate to both season of birth and breastfeeding. This should be explored further to better how much of the seasonal health differences can be explained by (and prevented with) differences in breastfeeding.

Despite the limitations listed above, the longitudinal aspect of the data offers many advantages. In particular, the data offer the ability to examine breastfeeding patterns while controlling for a number of covariates such as the mother’s education and labor force participation, income, marital status, maternal age, total number of children ever born, and the child’s birth order. The biggest contribution of this study is that it brings attention to the previously unexplored potential connection between season of birth, sex, health effects, and seasonality of breastfeeding in a developed nation.

## Conclusions

The findings of this study suggest that the costs and benefits of breastfeeding an infant vary with the season of birth and the sex of the child, even in a developed nation such as the US. Not only does the study find that seasonal variations for breastfeeding incidence and duration exist, but its findings also suggest that these seasonal variations differ by sex. This is consistent with the fact that many of the season of birth outcomes also differ by sex [2, 8, 10, 26].

Thus, seasonal differences in breastfeeding could explain some of the seasonal differences in health, personality, and other outcomes previously identified in the literature. Given these findings, it is imperative that future studies of breastfeeding seasonality consider potential differences in accordance with the child’s sex, while future examinations of season of birth effects should control for differences in breastfeeding. Subsequent studies should also examine the reasons for these seasonal differences in breastfeeding. For example, do work schedules or leisure patterns make it easier to breastfeed in one season or another? How does one’s specific climate affect breastfeeding decisions? Although lifestyle patterns and viral seasons tend to be similar for various geographic climates throughout the U.S., future research should analyze the impact of daylight hours, extreme temperatures, and geographical region on breastfeeding. Answering questions such as these is imperative for enabling policymakers and employers to best structure policies to reduce the risks associated with certain seasons of birth. Lastly, research linking seasonal effects of breastfeeding with diets in developing nations may be missing part of the picture. The existence of similar effects in the U.S., where malnutrition and starvation are less common, suggests other factors may be contributing to the observed effects.

## Data Availability

The National Longitudinal Survey of Youth is used for this study and is authorized under Title 29, Section 2, of the United States Code. The data described in this article can be freely and openly accessed at Investigator (nlsinfo.org) [33]. SAS programming code is available upon request.

## Abbreviations

EBF: Exclusive breast feeding
OLS: Ordinary least squares
WIC: Special Supplemental Nutrition Program for Women Infant and Children
